# Health risks of climate change in Australia: An umbrella review

**DOI:** 10.1016/j.joclim.2024.100347

**Published:** 2024-09-20

**Authors:** Michael Tong, Enembe Okokon, Sotiris Vardoulakis

**Affiliations:** a National Centre for Epidemiology and Population Health, The Australian National University, Canberra, ACT 2601, Australia; b Healthy Environments and Lives (HEAL) National Research Network, Australia; c HEAL Global Research Centre, Health Research Institute, University of Canberra, Bruce, ACT 2617, Australia

**Keywords:** Climate change, Health risks, Public health, Health outcomes, Risk assessment

## Abstract

**Introduction:**

The impact of climate change on population health has been extensively studied in Australia, but no comprehensive review of the impact of climate change on health in Australia has been performed. This review summarizes the most up-to-date, high-level evidence exploring the health risks of climate change in Australia, identifies evidence gaps in the scientific literature, and lays the groundwork for an in-depth national climate change and health risk assessment in Australia.

**Methods:**

Electronic database PubMed was searched for systematic reviews of the impact of climate change on health outcomes in Australia. Narrative synthesis was conducted to summarize findings.

**Results:**

The most frequently reported climate change related risks in Australia were heat and bushfires, followed by floods and droughts, with a limited number of studies on cyclones and rising sea levels. The impacts on health included all-cause mortality and morbidity, heat-related illnesses, vector-, food- and water-borne diseases, negative mental health effects, cardiovascular, respiratory, and renal diseases, injuries and adverse birth outcomes. These impacts were unevenly distributed across Australia's geographical regions and population groups, particularly affecting young children, people with health conditions or disabilities, the elderly, and pregnant women. There were notable gaps concerning First Nations, culturally and linguistically diverse groups, ethnic minorities, and refugees in the context of climate change and health in Australia.

**Conclusions:**

Further research is needed to deepen our understanding of the associations between climate change and health outcomes in Australia, especially among disadvantaged communities and sensitive population groups. Future risk assessments using standardized methodologies to estimate exposure-response functions for specific health outcomes are warranted. At-risk populations need to be adequately supported by a national adaptation plan that will reduce their vulnerability to climate extremes and prevent adverse health impacts of climate change in Australia.

## 1 Introduction

Climate change is posing a wide range of direct and indirect risks to population health and wellbeing, and to the infrastructure and functioning of the health system, some of which are well-recognised by the international public health community. Globally, the annual average temperature has increased by approximately 1 °C since 1880 [1]. The global temperature is projected to increase by about 1.5 °C by 2050 and 2–4 °C by the end of this century [2]. In Australia, the annual average surface temperature has increased by 1.4 °C since 1910 [2]. The projected temperature increases in Australia are higher than the global average by the middle and the end of this century [2]. According to the Intergovernmental Panel on Climate Change (IPCC) Sixth Assessment Report, Australia will experience a range of direct and indirect climate change impacts on its communities and environment, including more frequent and severe extreme weather events [2].

Climate change impacts have a profound effect on population health and wellbeing in Australia [3]. The rising average temperatures and increasing frequency and intensity of heatwaves have significantly increased all-cause mortality and morbidity [4,5]. The rising frequency and prolonged duration of bushfires associated with increasing temperature and droughts have also caused a pronounced impact on population health, particularly during the 2019–20 Black Summer bushfires, which resulted in more than 400 deaths attributable to bushfire smoke, more than 3,100 hospital admissions, more than 1,300 emergency department visits, 3 billion of animals killed or displaced, and more than 24 million hectares of lands scorched [6,7].

Climate change has increased health risks and generated adverse health effects, including all-cause mortality, morbidity and cardiorespiratory conditions [[8], [9], [10]]. Climate change related extreme weather events such as heatwaves, bushfires, floods, storms and droughts are occurring with greater frequency in Australia compared to half a century ago, and have yielded severe adverse health outcomes, including deaths, mental health issues, and injuries, particularly affecting socioeconomically disadvantaged groups and people living with chronic illness or disability [[10], [11], [12]].

These impacts of climate change on health have spurred a substantial demand on the health system, such as the need for emergency health services to address the immediate impacts of heatwaves, bushfires, floods, and storms, and healthcare services for the treatment of acute and chronic health conditions exacerbated by climate change [[13], [14], [15]].

Umbrella reviews are systematic collections and assessments of multiple systematic reviews and meta-analyses on a specific research topic [16]. Given the number of systematic reviews and meta-analyses published on the impact of climate change on health outcomes, an umbrella review provides an appropriate and informative approach for synthesizing the existing evidence on the topic. Therefore, the purpose of this umbrella review, in the context of the first National Climate Risk Assessment [17], is to summarize the most up-to-date, high-level evidence exploring the health risks of climate change in Australia, and identify evidence gaps in the peer-reviewed scientific literature. These findings lay the groundwork for an in-depth national climate change and health risk assessment in Australia.

## 2 Methods

### 2.1 Search strategy

This umbrella review identifies, catalogues, and summarizes the most up-to-date high-level evidence from published peer-reviewed systematic reviews reporting impacts of climate change related risks on health outcomes in Australia. It follows the Preferred Reporting Items for Systematic Review and Meta-Analysis (PRISMA) guidelines [18] and covers the period 2013–2023. The electronic database PubMed was searched for relevant information. Manual searches from the reference lists of selected articles were also performed to maximize retrieval of relevant reviews.

The search strategy included terms that combined climate change, health and social support in Australia, such as “climate change”, “global warming”, “hot temperature”, “heatwave”, “flood”, “drought”, “bushfire”, “extreme weather”, “health”, “disease”, “illness”, “death”, “social support”, “social service”, “health service”, “aid” and “assistance”. The detailed search terms are presented in Table S1.

### 2.2 Study selection and criteria

Reviews were included if the following criteria were met: 1) they explicitly described the impact of climate change on at least one health outcome in Australia, 2) full-text peer-reviewed systematic reviews (including scoping systematic reviews) written in English, and 3) published in the past 10 years (2013–2013). Studies were excluded for the following reasons: 1) lack of specific focus on climate change and health, 2) studies that were not systematic reviews, or 3) studies that were conducted in other countries without covering Australia as part of their study region.

### 2.3 Literature screening, data extraction and synthesis

The citations identified from the search strategy were first imported into the online literature review tool Covidence [19] for the title, abstract and full-text screening by two reviewers. Disagreements were resolved by group discussion, and a final decision was reached among the reviewers. In the following stage, a structured data collection sheet was developed to extract data from each review, and data were extracted from the included systematic reviews, such as author names, year, study site, search databases, study population, climate exposure, and health outcome. Data extraction was conducted by the first author and then verified by a second author, with a focus on the impact of climate change on health outcomes in Australia. A summary of the included systematic reviews is presented in Table S2. Findings were categorized into six climate change related health risks, and synthesized narratively to assess their impacts on health outcomes in Australia.

### 2.4 Quality and strength of evidence

The quality and strength of evidence were assessed using a modified version of the National Institutes of Health (NIH) Quality Assessment Tool for Systematic Reviews and Meta-Analyses [20]. The tool evaluates the validity of a study including focused questions, eligibility criteria, literature search, screening process, publication bias and heterogeneity. The strength of evidence was assessed based on the overall quality of evidence, and direction of the effect, other compelling characteristics of data that may influence certainty, and collective discussion within group.

## 3 Results

### 3.1 Study selection and inclusion

**Fig. 1:**
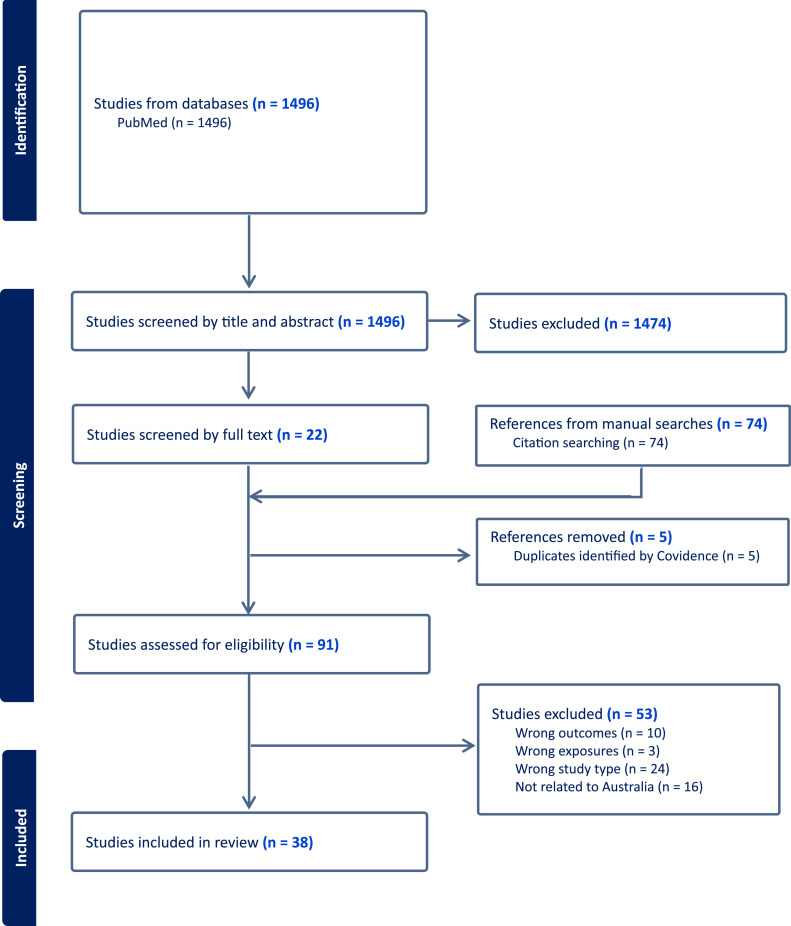
PRISMA flowchart for the literature search and selection process.

The literature search generated 1,496 records that were imported into Covidence for screening. Of these, 1,474 records were excluded from screening by title and abstract, resulting in 22 articles for full-text screening. There were 74 additional articles found from manual searches of the reference lists of 22 selected articles in order to maximize retrieval of relevant reviews, from which 5 duplicated records were removed. In total, 91 articles were assessed for eligibility, with 53 articles being further excluded after the assessment. Finally, 38 articles were included in this umbrella review. The study selection process is depicted in the flowchart (Fig. 1). It is important to note that not all of the articles included in this review focused solely on climate change-related health outcomes specifically in Australia. Some of the included articles also covered other regions.

### 3.2 Study context and characteristics

The characteristics of the included reviews and their key findings are summarised in Table S2. Most (33 out of 38 studies) studies were conducted at the global level with Australia as one of the study sites. There were 5 studies that specifically focused on Australia. The majority (31 out of 38) of the study populations were the general public including various sub-groups, with fewer reviews specifically focused on farmers (2 studies), women (2 studies) and children (3 studies).

The impact of climate change was categorized into six climate change related health risks including heat, bushfires, floods, droughts, cyclones, and rising sea levels on population health. The most frequently studied climate change-related exposures were heat (24 studies), followed by bushfires (8 studies), floods (4 studies), droughts (4 studies), cyclones and rising sea levels (1 study). The most frequently reported health outcomes were all-cause mortality and morbidity, heat-related illnesses, mental health effects, cardiovascular diseases, and respiratory diseases, followed by vector-borne diseases, renal diseases, injuries, food-borne and water-borne diseases, and adverse birth outcomes.

### 3.3 The impact of climate change related risks on health outcomes

The majority of included studies systematically reviewed climate change-related impacts—heat, bushfires, floods, and droughts—on population health. There was only one systematic review about the impact of cyclones and rising sea levels on population health. Table 1 illustrates the number of studies exploring climate change-related risks and associated health outcomes reported in Australia.

**Table 1:** Number of systematic review studies focusing on climate change related risks and health outcomes in Australia.

		Health outcomes										
		All-cause mortality/morbidity	Heat-related illnesses	Vector-borne diseases	Food-borne diseases	Water-borne diseases	Mental health disorders	Cardiovascular diseases	Respiratory diseases	Renal diseases	Injuries	Adverse birth outcomes
Climate change related risks	Heat	8	8	5	2	1	5	9	5	6	2	2
	Bushfires (smoke)	3	–	–	–	–	3	4	6	–	1	1
	Floods	1	–	2	1	1	3	1	1	1	1	1
	Droughts	–	–	–	–	–	4	–	–	–	–	–
	Cyclones	–	–	–	–	–	1	–	–	–	–	–
	Rising sea levels	–	–	–	–	–	1	–	–	–	–	–

#### 3.3.1 Heat and heatwaves

Impacts of heat on population health were reported in 24 reviews as the main consequence for population health in relation with climate change. The reported impacts of heat on population health outcomes included all-cause mortality and morbidity, heat-related illnesses, cardiovascular diseases, renal diseases, mental health disorders, respiratory diseases, vector-borne, food-borne and water-borne diseases, unintentional injuries, and adverse birth outcomes.

Specifically, a systematic review by Xu et al. including 14 original studies from Australia showed a 7 % increase in all-cause morbidity for each 5 °C increase in temperature [21]. Faurie et al. reviewed global studies including 8 studies from Australia and found heat-related illness morbidity and mortality increased by 18 % and 35 % for each 1 °C increase in temperature [22].

Damtew et al. reviewed global dengue studies including 3 studies from Australia and found the overall risk of the vector-borne disease was 1.13 for each 1 °C increase in temperature [23] and they also reviewed 17 Ross River Virus infection studies from Australia and found the relative risk between high temperature and the vector-borne disease was 1.09 per 1 °C increase [24]. Liu et al. reviewed global studies including 9 studies from Australia, and reported for each 1 °C increase in temperature, mental health-related mortality and morbidity increased with a RR of 1.022 and 1.009, respectively [25]. Phung et al. reviewed global studies including 10 studies from Australia and reported an increased risk of cardiovascular hospitalization of 2.2 % for heatwave exposure, but no association was observed for heat exposure [26].

Cheng et al. reviewed global studies including 8 studies from Australia, and found the heatwaves were not associated with an increased risk of cardiovascular diseases and respiratory diseases in Australia, even though significant associations were identified at the global level [27]. Liu et al. reviewed global studies including 23 studies from Australia and found each 1 °C increase in temperature, the risk of kidney-related morbidity increased by 1 % [28].

Fatima et al. reviewed global studies including 7 studies from Australia and showed the overall risk of occupational injuries also increased by 1 % for each 1 °C increase in temperature, and 17.4 % during heatwaves [29]. Chersich et al. reviewed global studies including 7 studies from Australia, and found higher temperature was associated with stillbirth and preterm birth in Australia [30].

#### 3.3.2 Bushfires

Impacts of bushfires on population health were reported in 8 reviews as another key concern in Australia. Several systematic reviews have highlighted the impact of bushfires (smoke) on all-cause mortality and morbidity, mental health disorders, cardiovascular diseases, injuries and adverse birth outcomes. This is especially pronounced in the context of the discernible influence on respiratory diseases. To be specific, Liu et al. reviewed global studies including 15 studies from Australia and found respiratory diseases were the most frequently reported health conditions associated with bushfires, and had the most consistent results [31]. Cardiovascular diseases and all-cause mortality were also frequently reported associated with bushfires [31,32].

Arriagada et al. reviewed global studies including 7 studies from Australia and found bushfire PM_2.5_ levels were positively associated with asthma hospitalizations and emergency department visits in Australia [33]. Henry et al. reviewed global studies including 3 studies from Australia and further showed bushfires would significantly increase respiratory emergency department visits and asthma hospitalizations within the first 3 days of exposure, particularly among children less than 5 years old [34]. Further, these systematic reviews showed children and the elderly were more vulnerable to bushfire PM_2.5_ [[32], [33], [34]].

Barros et al. reviewed 5 studies from Australia and found there were significant short-term health impacts of bushfires including both emergency department visits and hospital admissions for all-cause mortality, cardiovascular diseases and respiratory diseases [35]. However, Gao et al. reviewed 11 studies from Australia focusing on the long-term impacts of bushfire exposure on health and found mental health as the predominant issue, while the impacts on other health outcomes limited [36]. Zhang et al. also reviewed 5 Australian studies and found a pooled prevalence of 14 % for psychological distress in the general population at 2–4 years post bushfires [37], which would further support the long-term impact of bushfires on mental health in Australia. In addition, Evans et al. and Liu et al. found bushfires increased the incidence of pre-term/post-term birth outcomes, abnormal birth weight, and injuries [31,38].

#### 3.3.3 Floods, storms and extreme precipitation events

Impacts of floods**,** storms and extreme precipitation events on population health were reported in 4 reviews, particularly focusing on the impacts of climate change on mental health, vector-borne and water-borne diseases. For example, Dorji et al. reviewed global studies including 7 studies from Australia and concluded that climate change and associated flood events led to mental stress decreasing community wellbeing [39]. Fernandez et al. also indicated exposure to floods is associated with mental health problems including post-traumatic stress disorder, anxiety and depression in Australia [40]. Other studies found there were positive associations between flooding and vector-borne and water-borne diseases [41,42]. Particularly, Tall et al. reviewed 22 Australian studies and found for the Murray River, river flow and height were significantly associated with Ross River Virus (RRV) transmission and outbreaks [42]. More flood events in the context of climate change would cause increased mental health issues, vector-borne and water-borne disease outbreaks in Australia.

#### 3.3.4 Droughts

Impacts of droughts on population health were reported in 4 reviews in Australia and were the most frequently reported concern related to mental health issues. Specifically, Yazd et al. reviewed 29 Australian studies and reported that climate variability, particularly droughts in the context of climate change is the key risk factor affecting farmers’ and farm-workers’ mental health [43]. Further, another systematic review indicated an annual decrease in precipitation of 300 mm and exposure to droughts increased 8 % of the long-term mean suicide rate in Australia [44]. Batterham et al. reviewed 17 Australian studies and showed similar findings of a significant impact of droughts on mental health in Australia, which may persist beyond the drought period [45].

#### 3.3.5 Cyclones and rising sea levels

Compared with other climate change-related risks, systematic reviews on the impact of cyclones and rising sea levels on population health were less frequently reported in Australia. Dorji et al. reviewed global studies, including 7 studies from Australia, regarding climate change including cyclones and rising sea levels on population health, and identified adverse mental health effects as a key concern, particularly among rural and remote communities and First Nations peoples [39].

### 3.4 At-risk populations

At-risk populations in the context of climate change are quite diverse, including children, infants, the elderly, people with existing health conditions or disabilities, people of low socioeconomic status, women, pregnant women, people from First Nations, people from culturally and linguistically diverse backgrounds, people living in tropic and subtropical climate zones, farmers, people living in rural and remote areas and flood plains, outdoor workers, people exercising outdoors or practising sports, young people, and migrants. Table 2 illustrates these at-risk populations in the context of climate change related to each specific health outcome.

**Table 2:** At-risk populations and associated health outcomes exacerbated by climate change in Australia.

		Health							
		All-cause mortality/morbidity	Heat-related illnesses	Mental health disorders	Cardiovascular diseases	Respiratory diseases	Renal diseases	Injuries	Adverse birth outcomes
Climate change related risks	Heat	Children, Infant, Elderly, Pre-exsiting health condtions, Low SES [[46], [47], [48]]	Children,Elderly, Low SES, Pre-existing health conditions, Outdoor workers, Pregnant women, Ethnic minorities, [22,27,30,46]	Elderly, Pre-existing health conditions, Low SES, Tropical and subtropical climate zones [25,49]	Elderly, Pre-existing health conditions, Low SES, Tropical climate zones [27,46,50,51]	Children, Elderly, Pre-existing health conditions [27]	Elderly, Outdoor workers, Temperate climate zones [28]	Young workers, Migrant workers, Outdoor workers, Elderly [29,46]	Pregnant women [30]
	Bushfires (smoke)	Pre-existing health conditions, Less educated, Young adults aged<45 years, Elderly, Low SES [35,36]	–	–	Middle-aged and older adults [31]	Females, Elderly, Children, Pre-existing health conditions [31,33,34]	–	Pregnant women [38]	–
	Floods	Low SES, Pre-existing health conditions [41]	–	Children, Women [41]	–	Children [41]	–	–	–
	Droughts	–	–	Farmers, Rural communities, Children, Elderly, Women, Single parent [[43], [44], [45]]	–	–	–	–	–
	Cyclones	–	–	–	–	–	–	–	–
	Rising sea levels	–	–	–	–	–	–	–	–

Heat significantly increased risk of all-cause mortality and morbidity among children, infant, elderly, people with pre-existing health conditions, and people in low socioeconomic status [[46], [47], [48]]; heat-related illnesses among children, elderly, outdoor workers, people in low socioeconomic status, people with existing health conditions, pregnant women, and people from culturally and linguistically diverse backgrounds [22,27,30,46]; mental health issues among elderly, people with pre-existing health conditions, people in low socioeconomic status, people in tropical and subtropical climate zones [25,49]; cardiovascular diseases among elderly, people with pre-existing health conditions, people in low socioeconomic status, people in tropical climate zones [27,46,50,51]; respiratory diseases among children, elderly and people with pre-existing health conditions [27]; renal diseases among elderly, outdoor workers, and people in temperate climate zones [28]; injuries among elderly, young workers, migrant workers, and outdoor workers [29,46]; and adverse birth outcomes among pregnant women [30].

Bushfires significantly increased all-cause mortality and morbidity among people with pre-existing (respiratory) health conditions, less educated people, young adults aged <45 years, the elderly, and people in low socioeconomic status [35,36]; cardiovascular diseases among middle-aged and older adults [31]; respiratory diseases among children, elderly, females and people with pre-existing health conditions including asthma [31,33,34]; and adverse birth outcomes among pregnant women [38].

Floods significantly increased all-cause mortality and morbidity among people of low socioeconomic status, people with pre-existing health conditions [41]; mental health issues among children and women [41]; respiratory diseases among children [41]; and increased risk of injuries, vector-borne, food-borne and water-borne diseases during and after flooding in the general population.

Droughts increased the risk of mental health issues and suicides among farmers, children, the elderly, women, single parent and people living in rural and remote areas [[43], [44], [45]].

### 3.6 Quality and strength of evidence

Of the 38 studies included in this umbrella review, 22 studies were of “Good” quality and 16 were of “Fair” quality. The main reason for downgrading study quality to “Fair” was the ambiguity in the methods used in the review process. The overall quality of the evidence was rated as “Good”. The summary table of evidence grading is available in Supplementary Materials Table S3. The strength of evidence is presented in Supplementary Materials Table S4.

## 4 Discussion

Based on the evidence from the 38 systematic reviews included, the most significant climate change related health risks in Australia were associated with heat and bushfires, followed by floods and droughts, with fewer studies exploring the impact of cyclones and rising sea levels on population health.

Heat was extensively studied in Australia and mainly covered state and territory capital cities (Adelaide, Brisbane, Canberra, Darwin, Hobart, Melbourne, Sydney, and Perth) [21,52]. The findings of these systematic reviews indicated extreme heat significantly increased all-cause mortality and morbidity, particularly among the elderly, children, outdoor workers, people in low socioeconomic status, and people with existing health conditions. Further, extreme heat also increased the risk of certain categories of diseases including heat-related illnesses, mental health disorders, cardiovascular diseases, respiratory diseases, renal diseases, injuries, vector-borne, and food-borne and water-borne diseases.

Extreme heat was disproportionately associated with higher risks for at-risk populations. For example, the elderly were found to be at higher risk of cardiovascular diseases due to extreme heat. This higher risk could be attributed to the reduced thermoregulatory capacity among the elderly population, their inability to enhance blood circulation for effective body core temperature cooling, and subsequently caused acute and chronic impairments in sensitive bodily systems, resulting in the manifestation of cardiovascular diseases in extreme heat environments [50].

Outdoor workers, such as those employed in agriculture, forestry, and fishing were found to be at higher risk of injuries and renal diseases compared to other population groups due to climate change. This could be due to outdoor workers, mostly engaged in strenuous physical activities, and direct exposure to heat and solar radiation. These workers were often without protective measures, such as adequate hydration, or access to a shaded or air-conditioned environment. This lack of protective measures exacerbated their thermal stress, posing challenges to body thermoregulatory function in maintaining core body temperatures for optimal health, consequently leading to adverse conditions, such as loss of concentration, reduced vigilance and fatigue, and ultimately increased injuries and renal diseases [28,29].

Bushfires were reported as a significant concern in Australia in the context of climate change, with fire services responding to >45,000 bushfires in Australia every year [53]. Findings from these systematic reviews indicated bushfires significantly increased all-cause mortality and morbidity of respiratory diseases, cardiovascular diseases, and mental health effects in Australia [[31], [32], [33],^35^,^37^]. These outcomes were mainly due to exposure to bushfire smoke, which comprises air pollutants such as PM_2.5_, PM_10_, black carbon, carbon monoxide, and nitrogen oxides. A relatively smaller number of deaths and injuries was attributed to direct contact with fires.

Bushfire smoke can irritate the respiratory tract, trigger immune system responses, cause inflammation and oxidative stress in the airways and lungs, and potentially contribute to the development of chronic respiratory conditions [54,55]. Further, the inhalation of smoke particles may trigger a systemic inflammation response syndrome, which could increase systemic vascular permeability and oxidative stress, increasing the risk of cardiovascular events [56]. Moreover, people who experienced bushfire disasters may suffer from trauma, loss of property, displacement and stress, which are likely to increase the likelihood of negative mental health outcomes [37,57].

Most reviewed studies identified population groups at higher risk of mortality and morbidity due to bushfires, including people living with pre-existing health conditions, children, young and elderly adults, women and particularly pregnant women. People with pre-existing health conditions, such as asthma, or disabilities, young children and the elderly were more susceptible to the harmful effects of bushfire smoke [31,33,34,36]. This could be due to the fact that they have more sensitive airways and relatively weaker lung function [31,34].

A number of reviews indicated floods will increase the risk of all-cause mortality and morbidity, vector-borne, food-borne, and water-borne diseases, mental health disorders, cardiovascular diseases, respiratory diseases, renal diseases and injuries. Both floods and droughts have significantly increased the risk of mental health problems in Australia [39,40,43,45]. People who witness these extreme events may experience property damage, loss of livelihoods and possessions, displacement from homes, uncertainty and fear about the future. These feelings and emotions can lead to traumatic stress and anxiety, disruption of social networks, loss of a sense of belonging, and ultimately mental health disorders [41,43].

The vulnerability of populations can extend to people living in rural and remote areas, particularly to rural residents with pre-existing health conditions or disabilities, young children, the elderly, and women who have the greatest need for healthcare and social support services. The vulnerability of people living in rural and remote areas may be attributed to the geography, with certain rural areas in Australia, such as Lismore, Gippsland and much of Western Australia, being more flood or drought prone. Rural and remote areas have relatively limited access to healthcare, experience delayed medical care and on occasions disrupted primary and secondary healthcare services, making them more susceptible to the health impacts of climate related extreme events [43,45]. Moreover, people with pre-existing health conditions or disabilities, children, the elderly, and pregnant women living in rural areas may have reduced physiological resilience, developing or weakened immune systems, and heightened sensitivity to psychological effects of extreme weather events inducing stress, anxiety, and depression, thereby increasing their vulnerability to health issues [41,44]. These at-risk groups are in need of more social support, such as healthcare services, housing assistance and financial aid to increase their resilience to climate change. In addition, First Nations, culturally and linguistically diverse groups, ethnic minorities, and refugees, need more social support to address health challenges in the context of climate change.

Limitations of this umbrella review should be acknowledged. It only included systematic reviews published within a ten-year period (2013–2023). As such, emerging evidence from recent rapid reviews (e.g., Lee et al. [58]) was not included. Records were only searched in one database (PubMed). Moreover, this review did not include the indirect impacts of climate change on health, such as those through its effects on housing, the economy, and infrastructure, which subsequently influence health outcomes.

### Future research recommendations and implications for public health

Evidence on the impact of climate change on population health is well established in Australia, particularly in relation to heat. Notably, reviews focusing on heat and health exhibit a higher level of quality compared to those addressing other aspects. However, due to diverse landscapes and climate zones, and heterogeneity in study findings in Australia, population-based high-quality evidence with quantitative analysis of climate change related risks is still limited. Further, evidence on other climate change-related risks such as floods, droughts, cyclones and rising sea levels on population health is needed. Additionally, it is worth noting that many reviews concentrate on general health outcomes such as all-cause mortality or morbidity. However, there is a recognized gap in research regarding specific health outcomes that have not been sufficiently studied, such as renal diseases, mental health disorders, food-borne and water-borne diseases.

The distribution of health impacts from climate change is disproportionately experienced by at-risk populations, such as young children, people with health conditions or disabilities, the elderly, and pregnant women in Australia. Further understanding of how climate change impacts the health of these at-risk populations, and tailored interventions to address their specific health challenges arising from climate change, can facilitate the development of appropriate responses to climate change preparedness and adaptation for at-risk populations in Australia in the context of climate change. These tailored interventions should also address health disparities in access to healthcare services. Importantly, more systematic exploration is needed of the disproportional health burden, compounded by climate change, affecting First Nations in Australia, as well as of the disruption to their adaptive capacity caused by European colonization [59].

Further, literature on the health effects of climate change in remote and rural areas of Australia was limited compared to urban areas in capital regions, making it an urgent requirement to conduct more studies in under-investigated rural and remote areas of Australia.

Future well-designed studies using a standardized methodology to estimate risk coefficients for different health outcomes across both urban and rural areas of Australia, particularly focusing on at-risk population groups, including First Nations, culturally and linguistically diverse groups, ethnic minorities, and refugees, are needed to fill current evidence gaps. Moreover, intersectoral collaboration can facilitate the development of public health interventions in the face of climate change. Future research that is policy or action oriented, such as co-benefit analysis to inform policy changes or community-based adaptation research involving co-design and co-production, is also warranted.

## 5 Conclusion

The impacts of climate change on health are already occurring. Climate change related risks and extreme weather events in Australia will continue to increase in duration and frequency due to the effects of climate change. This umbrella review highlights the impacts of climate change on population health across various areas in Australia, particularly in relation to the impacts of extreme heat on all-cause mortality and morbidity, heat-related illnesses, cardiovascular diseases and respiratory diseases. Further, the health effects of other climate change related extreme events, including bushfires, floods and droughts, on mental health were explored in a number of studies in Australia. However, published studies have not yet provided a comprehensive assessment of the full extent of climate change impacts on specific health outcomes in Australia. High-quality evidence with quantitative analysis of climate change-related impacts on specific health outcomes for diverse population groups is still limited. Future well-designed studies considering standardized methodology to estimate risk coefficients for specific health outcomes and population groups are warranted to fill current knowledge gaps. More studies focusing on at-risk populations, First Nations, and other socioeconomically disadvantaged groups, and intersectional collaboration to develop effective health interventions are particularly needed.

## Ethical statement

No ethics required to carry out the review.

## Funding

This research is funded by the National Health and Medical Research Council Healthy Environments and Lives (HEAL) National Research Network, Grant no. 2008937.

## CRediT authorship contribution statement

**Michael Tong:** Writing – review & editing, Writing – original draft, Visualization, Validation, Project administration, Methodology, Investigation, Formal analysis, Conceptualization. **Enembe Okokon:** Writing – review & editing, Validation, Methodology, Formal analysis, Data curation. **Sotiris Vardoulakis:** Writing – review & editing, Validation, Methodology, Funding acquisition, Conceptualization.

## Declaration of competing interest

The authors declare that they have no known competing financial interests or personal relationships that could have appeared to influence the work reported in this paper.

## Data Availability

All relevant data within the manuscript are available in the original published journal articles.

## Appendix Supplementary materials

Image, application 1
