# Supplementary material for: Health risks of climate change in Australia: An umbrella review
Source: J Clim Chang Health. 2024 Sep 20;20:100347. doi: 10.1016/j.joclim.2024.100347 (PMC12851267; doi:10.1016/j.joclim.2024.100347)
Supplement: Supplementary file 1 [file mmc1.docx]

**Appendix: Supplementary Materials to**

**Title: Health risks of climate change in Australia: An umbrella review**

**Author List and Affiliations:**

Michael Tong ^1,2*^

Enembe Okokon ^2,3^

Sotiris Vardoulakis ^2,3^

1. National Centre for Epidemiology and Population Health, The Australian National University, Canberra, Australian Capital Territory 2601, Australia. E-Mail: [Michael.Tong@anu.edu.au](mailto:Michael.Tong@anu.edu.au) (MT)
2. Healthy Environments And Lives (HEAL) National Research Network, Australia
3. HEAL Global Research Centre, Health Research Institute, University of Canberra, Bruce, Australian Captial Territory 2617, Australia. Email: [Enembe.Okokon@canberra.edu.au](mailto:Enembe.Okokon@canberra.edu.au) (EO); [Sotiris.Vardoulakis@canberra.edu.au](mailto:Sotiris.Vardoulakis@canberra.edu.au) (SV)

**Table S1.** Search terms used to identify literature regarding the impact of climate change related risks on health outcomes in Australia

| **Key concept** | **Keywords** |
| --- | --- |
| Climate change | (climat* change OR global warm* OR sea level ris* OR hot temperature* OR high temperature* OR cyclonic storms OR drought* OR flood* OR heavy rain* OR extreme rain* OR natural disaster* OR wildfire* OR bushfire* OR extreme weather* OR air pollut*) |
| Health | (health* OR disease* OR morbidity OR mortality OR death* OR heat related illness* OR heat related disease* OR infectious disease* OR vector borne disease* OR mosquito borne disease* OR food borne disease* OR water borne disease* OR mental health* OR renal disease* OR kidney disease* OR cardiovascular disease* OR respiratory disease* OR injury OR injuries) |
| Social support | (health service* OR healthcare service* OR social service* OR social support* OR social assistance* OR housing assistance* OR housing support* OR financial aid* OR financial support* OR financial assistance* OR emergency service* OR emergency support* OR emergency assistance* OR healthcare facilit* OR healthcare system* OR hospital* OR infrastructure* OR community health* OR general practice* OR public health* OR population health* OR workforce* OR supply chain* OR transport* OR ambulance*) |
| Australia | (Australia* OR Oceania* OR Oceanica* OR New South Wales OR NSW OR Victoria OR Queensland OR Northern Territory OR Western Australia OR South Australia OR Tasmania OR Australian Capital Territory OR Torres Strait Island OR Canberra OR Sydney OR Melbourne OR Brisbane OR Darwin OR Perth OR Hobart OR Adelaide OR Newcastle OR Wollongong OR Gold Coast OR Cairns OR Townsville) |

* Retrieve unlimited suffix variations.

**Table S2.** Summary of study characteristics and key findings

| **Study ID** | **Author, year** | **Study site** | **Search databases (# of articles in review)** | **Study population (Vulnerable population)** | **Climate-related exposure** | **Health outcome** | **Key findings** |
| --- | --- | --- | --- | --- | --- | --- | --- |
| 1 | Adnan M, et al., 2022 [1] | Australia (Overall) | Web of Science, Scopus, PubMed (107) | The general population (outdoor workers, elderly, people with pre-existing health conditions, low SES) | Heat | Mortality, heat-related mortality, mental health, cardiovascular diseases, renal disease, work-related injuries, adverse birth outcomes | The frequency, severity and duration of heatwaves in Australian cities have been increasing and are likely to continue under climate change scenarios. Heatwave vulnerability is associated with geographical and climatic factors, space, time, socioeconomic and demographic factors, as well as the physiological condition of people. |
| 2 | Alderman K, et al., 2012 [2] | Global including Australia | Medline, Proquest, Informit, ScienceDirect (35) | The general population | Flood | Mortality, vector-, food- and water-borne diseases, mental health, cardiovascular diseases, respiratory diseases, renal diseases, injuries, adverse birth outcomes | Mortality rates were found to increase by up to 50% in the first-year post-flood. After floods, it was found there is an increased risk of disease outbreaks, particularly in areas with poor hygiene and displaced populations. Psychological distress in survivors (prevalence 8.6% to 53% two years post-flood) can also exacerbate their physical illness. Global trends in urbanization, burden of disease, malnutrition and maternal and child health must be better reflected in flood preparedness and mitigation programs. |
| 3 | Barros B, et al., 2023 [3] | Global including Australia | Web of Science, ScienceDirect, Scopus, PubMed (52) | The general population | Bushfire | Mortality, cardiovascular diseases, respiratory diseases | Extremely high levels of pollutants, mostly of the fine fraction of particulate matter (PM) and ozone, were associated with intense bushfire emissions and reported to exceed the WHO guidelines by several-fold. Adverse health outcomes include emergency department visits and hospital admissions for cardiorespiratory diseases as well as mortality. Globally, a significant association was found for all-cause respiratory outcomes, but mixed results were noted for cardiovascular-related effects. For the latter, estimates were only significant several days after bushfire emissions, suggesting a more delayed impact on the heart. |
| 4 | Batterham P, et al., 2022 [4] | Global including Australia | PsycINFO, Medline, Web of Science (28) | Population in rural areas | Drought | Mental health | Poorer states of mental health and well-being were typically found to have an association with extreme climate or weather events and environmental degradation. |
| 5 | Arriagada BN, et al., 2019 [5] | Global including Australia | PubMed, Medline, Embase, Scopus (20) | The general population | Bushfire | Respiratory diseases | Fire smoke PM_2.5_  levels were positively associated with asthma hospitalizations (RR=1.06, 95% CI: 1.02–1.09) and emergency department (ED) visits (RR=1.07, 95% CI: 1.04–1.09). Subgroup analyses found that females were more susceptible than males for ED visits, and that there was an increasing association by age groups for hospital admissions and ED visits. High heterogeneity between studies was observed, but the results were robust to sensitivity analysis. |
| 6 | Campbell S, et al., 2018 [6] | Global including Australia (Brisbane, Sydney, Melbourne, Adelaide, Perth) | PubMed, Scopus, Web of Science, CINAHL (188) | The general population | Heat | Mortality, cardiovascular diseases | The population most at risk of death and illness from extreme heat is under-represented. The majority of studies examined mortality as a key indicator of population-wide impact, rather than the more sensitive indicator of morbidity. Heatwave and health impact research is needed in regions where the impact is most severe. |
| 7 | Cheng J, et al., 2019 [7] | Global including Australia (Adelaide, Brisbane) | PubMed, Scopus, Embase, Web of Science (54) | The general population (elderly, people with pre-existing health conditions) | Heat | Cardiovascular diseases, respiratory diseases | Globally, there were significant associations between heatwaves and cardiovascular mortality (RR): 1.149, 95% confidence interval (CI): 1.090, 1.210) and respiratory mortality (RR: 1.183, 95%CI: 1.092, 1.282). Heatwaves appeared to be marginally associated with cardiovascular and respiratory morbidities (RR: 0.999, 95%CI: 0.996, 1.002 for cardiovascular morbidity; RR: 1.043, 95%CI: 0.995, 1.093 for respiratory morbidity). For mortality, significant associations were observed for the elderly, ischemic heart disease, stroke, heart failure, and chronic obstructive pulmonary disease. Sensitivity analyses suggested that these findings were robust. However, heatwaves were not associated with increased risk in Australia. |
| 8 | Cheng J, et al., 2019 [8] | Global including Australia (Brisbane) | PubMed, Web of Science, Scopus (97) | The general population (elderly) | Heat | Mortality, heat-related illnesses cardiovascular diseases, food-borne diseases | Under the warming climate scenario, almost all projections assuming no population adaptation suggested a future increase in heat-related but a decrease in cold-related burden of disease. Heat-related attributable morbidity burden (5.9%) was greater than cold-related (1.5%) in Australia. Heat was associated with increased years of life lost from all-cause disease and cardiovascular disease in Australia. Future temperature rise would lead to an increase in years of life lost due to disability from salmonella infection in Australia by 2030 and 2050. |
| 9 | Chersich M, et al., 2020 [9] | Global including Australia (Brisbane, Alice Springs) | Medline, Web of Science (70) | Women | Heat | Adverse birth outcomes | Odds of a preterm birth rose 1.05-fold (95% confidence interval 1.03 to 1.07) per 1°C increase in temperature and 1.16-fold (1.10 to 1.23) during heatwaves. Higher temperature was associated with reduced birth weight. Studies on stillbirths showed associations between temperature and stillbirth, with stillbirths increasing 1.05-fold (1.01 to 1.08) per 1°C rise in temperature. In Australia, stillbirths increased 1.46-fold (95%CI 1.09-1.96) per 1°C rise in temperature. Associations between heat and preterm birth were highest in indigenous women in coastal regions of Australia. Associations between temperature and outcomes were largest among women in lower socioeconomic groups and at age extremes. Temperature rises with global warming could have major implications for pregnancy outcomes. |
| 10 | Yazd S, et al., 2019 [10] | Global including Australia | PsycINFO, PubMed, Scopus, Google Scholar (167) | Farmers | Drought | Mental health | The four most-cited influences on farmers’ mental health in the reviewed literature respectively were pesticide exposure, financial difficulties, climate variabilities / drought, and poor physical health/past injuries. In Australia, drought and climate change are key risk factors affecting farmers’ mental health. Psychological health disturbances were more common in farmers and farm-workers. |
| 11 | Damtew Y, et al., 2023 [11] | Global including Australia (Queensland, Cairns) | PubMed, Scopus, Embase, Web of Science (106) | The general population | Heat | Vector-borne diseases (Dengue) | The overall pooled estimate showed a 13% increase in risk of dengue infection (RR = 1.13; 95% confidence interval (CI): 1.11–1.16) for each 1 °C increase in high temperatures. Subgroup analyses by climate zones suggested greater effects of temperature in tropical monsoon climate zone (RR = 1.29, 95% CI: 1.11–1.51) and humid subtropical climate zone (RR = 1.20, 95% CI: 1.15–1.25). Heatwave events showed an association with an increased risk of dengue infection (RR = 1.08; 95% CI: 0.95–1.23), despite a wide confidence interval. |
| 12 | Damtew Y, et al., 2022 [12] | Australia (VIC, QLD, SA, NT, WA, NSW, TAS) | PubMed, Scopus, Embase, Web of Science (17) | The general population | Heat | Vector-borne diseases (RRV) | The overall RR for the association between temperature and the risk of RRV infection was 1.09 (95% confidence interval (CI): 1.02, 1.17). Subgroup analyses by climate zones showed an increase in RRV infection relative to temperature increase in humid subtropical and cold semi-arid climate zones. |
| 13 | Dorji T, et al., 2023 [13] | Global including Australia (North Australia, Victoria) | Scopus, Web of Science, ProQuest, Google Scholar (23) | The general population (Farmers, fishers, aboriginal people) | Climate change in general, heat, flood, drought, cyclone, rising sea level | General health, mental health | The study found climate change led to mental stress, decreasing community wellbeing. Common impacts on community wellbeing from climate change include heatwaves, rainfall (either drought or flood), atmospheric warming, cyclones and sea level rise. Climate change mitigation strategies should also include investment in public infrastructure like transport, energy and housing. |
| 14 | Evans J, et al., 2022 [14] | Global including Australia | Scopus, CINAHL, PubMed, Google Scholar (16) | Women | Bushfire | Adverse birth outcomes | The incidence of preterm/post-term birth and low birth weight in areas affected by fire in Australia was higher. Exposure to wildfire changes birth weight. Studies indicate that wildfire exposure may be associated with changes in birth outcomes and increased morbidity for childbearing women and their babies. These effects may be profound and have long-term and wide-ranging public health implications |
| 15 | Fatima S, et al., 2021 [15] | Global including Australia (Adelaide, Melbourne, Brisbane, Perth) | PubMed, Embase, Scopus (24) | The general population (young, male, outdoor workers, migrant workers) | Heat | Injuries | The overall risk of injuries increased by 1% (RR 1.010, 95% CI: 1.009–1.011) for 1 °C increase in temperature above reference values and 17.4% (RR 1.174, 95% CI: 1.057–1.291) during Heatwaves. Among different climate zones, the highest risk of injuries during hot temperatures was identified in Humid Subtropical Climates (RR 1.017, 95% CI: 1.014–1.020) followed by Oceanic (RR 1.010, 95% CI: 1.008–1.012) and Hot Mediterranean Climates (RR 1.009, 95% CI: 1.008–1.011). Similarly, Oceanic (RR 1.218, 95% CI: 1.093–1.343) and Humid Subtropical Climates (RR 1.213, 95% CI: 0.995–1.431) had the highest risk of injuries during heatwave periods. |
| 16 | Faurie C, et al., 2022 [16] | Global including Australia (Adelaide, SA, Sydney, NSW, Perth, Queensland, TAS) | PubMed, Embase, Scopus (62) | The general population (elderly) | Heat | Heat-related illnesses | For every 1 °C increase in temperature, the RR of direct heat illness morbidity and mortality increased by 18% and 35%, respectively. For morbidity, the greatest increase was for direct heat illness (RR 1.45, 95%CI: 1.38–1.53), compared to dehydration (RR 1.02, 95%CI: 1.02–1.03). There was a higher risk for people aged >65 years (RR 1.25; 95% CI: 1.20–1.30), and those living in subtropical climates (RR 1.25; 95% CI: 1.21–1.29). |
| 17 | Fernandez A, et al., 2015 [17] | Global including Australia | PubMed, Web of Science (83) | The general population | Flood | Mental health | The level of exposure to floods has been systematically associated with mental health problems. The main mental health disorders are post-traumatic stress disorder, depression and anxiety. Housing, financial losses, disruption to daily routines including temporary or permanent lost of employment, or loss of services as consequences of flood presented with higher levels of mental health problems. Social support was a protective factor for mental disorders. Low socioeconomic status is associated with poor mental health outcomes after exposure to floods. |
| 18 | Gao Y, et al., 2023 [18] | Global including Australia | Medline, Embase, Scopus (36) | The general population | Bushfire | Mortality, mental health, cardiovascular diseases, respiratory diseases | Studies predominantly focused on mental health (21 studies, 58.33%), while evidence on the long-term impacts of wildfire exposure on health outcomes other than mental health is limited. Current evidence indicated that long-term impacts of non-occupational wildfire exposure were associated with mortality (COVID-19 mortality, cardiovascular disease mortality and acute myocardial disease mortality), morbidity (mainly respiratory diseases), mental health disorders (mainly posttraumatic stress disorder), shorter height of children, reduced lung function and poorer general health status. |
| 19 | Henry S, et al., 2021 [19] | Global including Australia | Medline, Embase, CINAHL, Greenfile, Web of Science, CABI, ProQuest, Scopus, HERO (16) | Children | Bushfire | Respiratory diseases | There is a significant increase in respiratory emergency department visits and asthma hospitalizations within the first 3 days of exposure to wildfire smoke, particularly in children < 5 years old. |
| 20 | Huang C, et al., 2011 [20] | Global including Australia | PubMed, Scopus, ScienceDirect, ProQuest, Web of Science (14) | The general population (elderly, people with heart disease) | Heat | Mortality, heat-related illnesses, | Most projections showed that climate change would result in a substantial increase in heat-related mortality. Further research is needed to provide a stronger theoretical framework for projections, including a better understanding of socioeconomic development, adaptation strategies, land-use patterns, air pollution, and mortality displacement. |
| 21 | Kolves K, et al., 2013 [21] | Global including Australia | Scopus, Medline, Web of Knowledge, ProQuest, Safetylit (42) | The general population | Drought | Mental health | Drought in Australia increased the likelihood of suicide. An annual decrease in precipitation of about 300mm would lead to an increase of approximately 8% in the long-term mean suicide rate. |
| 22 | Liu J, et al., 2015 [22] | Global including Australia | PubMed, Scopus (61) | The general population | Bushfire | Mortality, cardiovascular diseases, respiratory diseases, injuries | Daily pollution levels during or after wildfire in most studies exceeded U.S. EPA regulations. Levels of PM10, the most frequently studied pollutant, were 1.2 to 10 times higher due to wildfire smoke compared to non-fire periods and/or locations. Respiratory disease was the most frequently studied health condition, and had the most consistent results. The majority of studies found that wildfire smoke was associated with an increased risk of respiratory and cardiovascular diseases. Children, the elderly and those with underlying chronic diseases appear to be susceptible. |
| 23 | Liu J, et al., 2021 [23] | Global including Australia (Adelaide, Brisbane, Sydney, Perth, Cairns, Melbourne, QLD, NSW, TAS, WA) | PubMed, Embase, Scopus, Web of Science, CINAHL (91) | The general population (aged >64 years, males, elderly) | Heat | Renal diseases | With a 1 °C increase in temperature, the risk of kidney-related morbidity increased by 1% (RR 1.010; 95% CI: 1.009–1.011), with the greatest risk for urolithiasis. Heatwaves were also associated with increased morbidity with a trend observed with heatwave intensity. During low-intensity heatwaves, there was an increase of 5.9% in morbidity, while during high-intensity heatwaves there was a 7.7% increase. There were greater RRs for males, people aged ≤64 years, and those living in temperate climate zones. Similarly, for every 1 °C temperature increase, there was a 3% (RR 1.031; 95% CI: 1.018–1.045) increase in the risk of kidney-related mortality, which also increased during heatwaves. High temperatures (and heatwaves) are associated with an elevated risk of kidney disease outcomes. |
| 24 | Liu J, et al., 2021 [24] | Global including Australia (Adelaide, NSW, Sydney, QLD, Brisbane) | PubMed, Embase, Scopus, Web of Science, PsycINFO (53) | The general population | Heat | Mental health | For each 1 °C increase in temperature, the mental health-related mortality and morbidity increased with a RR of 1.022 (95%CI: 1.015–1.029) and 1.009 (95%CI: 1.007–1.015), respectively. The greatest mortality risk was attributed to substance-related mental disorders (RR, 1.046; 95%CI: 0.991–1.101), followed by organic mental disorders (RR, 1.033; 95%CI: 1.020–1.046). A 1 °C temperature rise was also associated with a significant increase in morbidities such as mood disorders, organic mental disorders, schizophrenia, neurotic and anxiety disorders. Findings suggest evidence of vulnerability for populations living in tropical and subtropical climate zones, and for people aged more than 65 years. This problem will likely increase with a warming climate, especially in the context of climate change. |
| 25 | Liu J, et al., 2022 [25] | Global including Australia (Brisbane, Sydney, Adelaide, Perth, NSW, TAS, QLD) | PubMed, Embase, Scopus (282) | The general population (low SES, elderly) | Heat | Cardiovascular diseases | A 1°C increase in temperature was positively associated with cardiovascular disease-related mortality. The overall risk of  cardiovascular disease-related mortality increased by 2.1%, with the highest specific disease risk being for stroke and coronary heart disease. A 1°C temperature rise was also associated with a significant increase in morbidity.  The findings suggest heat exposure leads to elevated risk of morbidity and mortality for women, people 65 years and older, individuals living in tropical climates, and those in countries of lower-middle income. Heatwaves were also significantly associated with 11.7% increase in risk of mortality. |
| 26 | Mason H, et al., 2022 [26] | Australia (Overall) | Medline, Scopus, Web of Science, PsychINFO, ProQuest, ScienceDirect (45) | The general population (children, elderly, low SES, people with existing health conditions) | Heat | Mortality, heat-related illnesses, cardiovascular diseases, respiratory diseases, renal diseases, mental health | Significant increases in mortality, as well as hospital, emergency, and ambulance demand, were found across Australia during heatwave periods. Admissions for cardiovascular, renal, respiratory, mental and behavioural conditions exhibited increases during heatwaves. The most vulnerable groups during heatwaves were children (< 18 years) and the elderly (60+). |
| 27 | Naish S, et al., 2014 [27] | Global including Australia (Overall) | PubMed, Scopus, ScienceDirect, ProQuest, Web of Science (16) | The general population | Heat | Vector-borne diseases | Most studies showed that the transmission of dengue is highly sensitive to climatic conditions, especially temperature, rainfall and relative humidity. Studies on the potential impacts of climate change on dengue indicate increased climatic suitability for transmission and an expansion of the geographic regions at risk during this century. |
| 28 | Phung D, et al., 2016 [28] | Global including Australia (Brisbane, Perth, Sydney, NT) | PubMed (64) | The general population (elderly, people aged >40 years with pre-existing CVD) | Heat | Cardiovascular diseases | The relative risk of cardiovascular hospitalization increased 2.8% (95% CI, 1.021–1.035) for cold exposure, 2.2% (95% CI, 1.006–1.039) for heatwave exposure, and 0.7% (95% CI, 1.002–1.012) for an increase in diurnal temperature. The significant dose–response relationship of temperature—cardiovascular admission was found with cold exposure and diurnal temperature. There is a significant short-term effect of cold exposure, heatwave and diurnal variation on cardiovascular hospitalizations. |
| 29 | Skinner R, et al., 2022 [29] | Global including Australia | OvidSP, Medline, DARE, CINAHL, PubMed, Scopus (21) | The general population | Bushfire (smoke) | Cardiovascular diseases, respiratory diseases | Literature regarding the relationship between emergency department (ED) presentations and wildfire events is primarily limited to studies from the United States and Australia and indicates particulate matter (PM) is principally linked to adverse respiratory and cardiovascular outcomes. Observable trends in the literature principally included a significant increase in respiratory presentations, primarily with a lag of one to two days from the initial event. Individuals under five, over 65, or those with pre-existing conditions formed the majority of ED presentations. |
| 30 | Tall J, et al., 2014 [30] | Australia | Medline, PubMed, CINAHL, ScienceDirect, JSTOR, Scopus, Web of Science (22) | The general population | Flood | Vector-borne diseases | Overall, the evidence to support a positive association between flooding and vector-borne disease (Ross River virus RRV)  outbreaks is largely circumstantial, with the literature mostly reporting only coincidental occurrence between the two. However, for the Murray River, river flow and height (surrogates of flooding) were positively and significantly associated with RRV transmission. More frequent flood events  arising from climate change may result in increased outbreaks of RRV disease. |
| 31 | Thompson R, et al., 2023 [31] | Global including Australia (Adelaide, Brisbane, Darwin, Sydney, Melbourne, Perth, Hobart, Canberra) | Web of Science, Embase, PsychINFO, PubMed (144) | The general population | Heat | Mental health | A 1°C increase in mean monthly temperature was associated with an increase in incidence of 1.5% (95% CI 0.8–2.2); a 1°C increase in mean daily temperature was associated with an increase in incidence of 1.7% (0·3–3·0). Heatwaves versus non-heatwave periods were associated with an increase in incidence of 9.7% (95% CI 7.6–11.9). Increased temperature and variability could be associated with increased cases of suicide and suicidal behaviour, hospital attendance or admission for mental illness. |
| 32 | Wu Q, et al., 2023 [32] | Global including Australia (Brisbane, Sydney) | PubMed, Web of Science (15) | The general population (elderly, males) | Heat | Cardiovascular diseases | Extreme heat was significantly associated with an increased risk of OHCA, and the pooled relative risk (RR) was 1.071 [95 % confidence interval (CI): 1.019–1.126]. The risk of OHCA was also elevated by heatwaves (RR = 1.248, 95%CI: 1.091–1.427) and more intensive heatwaves had a greater effect. The elderly and males seemed to be more vulnerable to the effects of heat. |
| 33 | Xu Z, et al., 2020 [33] | Global including Australia (Overall) | PubMed, ProQuest, ScienceDirect, Scopus, Web of Science (16) | The general population | Heat | Vector-borne diseases | Most studies reported an increase in disease burden, a wider spatial distribution of dengue cases or more people exposed to climatically suitable areas of dengue as climate change proceeds. The years 1961–1990 and 2050 were the most commonly used baseline and projection periods, respectively. Instead of projecting the future number of dengue cases, there is a growing consensus on “population exposed to climatically suitable areas for dengue” or “epidemic potential of dengue cases”. |
| 34 | Xu Z, et al., 2018 [34] | Global including Australia (Adelaide, Brisbane, Perth, Melbourne, Sydney, Canberra) | PubMed, ProQuest, ScienceDirect, Scopus (19) | Children | Heat | Respiratory diseases | Increasing evidence suggests a non-linear relationship between absolute temperature and childhood asthma. Both high and low temperatures were associated with an increase in emergency department admissions for asthma, and the relative risks at lag 0–1 days for heat and cold were 1.12 (95% CI 1.02, 1.22) and 1.45 (95% CI 1.20, 1.74), respectively. A large intra-day temperature variation (i.e. diurnal temperature range (DTR)) increased the occurrence of childhood asthma. |
| 35 | Xu Z, et al., 2012 [35] | Global including Australia (Adelaide, Sydney, Brisbane, Melbourne, Canberra) | PubMed, ProQuest, ScienceDirect, Scopus, Web of Science (33) | Children (under 1 year) | Heat | Mortality, heat-related illnesses, vector-borne diseases, food-borne diseases, water-borne diseases, respiratory diseases, renal diseases, | Very young children are particularly vulnerable to heat-related deaths. Hot temperatures mainly affect cases of infectious diseases among children, including gastrointestinal diseases, malaria, hand, foot and mouse diseases, and respiratory diseases. During heatwaves, the incidences of renal disease, fever and electrolyte imbalance among children increase significantly. Mortality rises among children aged 0-4 years in heatwaves, and significant rise of renal hospital admission in the 5–14 years age group during heatwaves in Australia. |
| 36 | Xu Z, et al, 2014 [36] | Global including Australia (Adelaide) | PubMed, ProQuest, ScienceDirect, Scopus, Web of Science (12) | Child (under 1 year) | Heat | Mortality, heat-related illnesses, respiratory diseases, renal diseases | The existing literature does not consistently suggest that mortality among children  increases significantly during heat waves, even though infants were associated with more heat-related deaths. Pediatric diseases or conditions associated with heatwaves include renal disease, respiratory disease, electrolyte imbalance and fever. Heatwaves had a significant impact on children’s mortality in Australia. |
| 37 | Xu Z, et al., 2023 [37] | Global including Australia (Adelaide, Brisbane, Canberra, Darwin, Perth, Sydney, Melbourne, Hobart NSW, TAS) | PubMed, Embase, CINAHL, Scopus, ProQuest, Web of Science (48) | The general population | Heat | Mortality, heat-related illnesses, cardiovascular diseases, renal diseases | For each 5 °C increase in mean temperature, the risk of ambulance dispatches for all causes and for cardiovascular diseases increased by 7% (95% confidence interval (CI): 5%, 10%) and 2% (95% CI: 1%, 3%), respectively, but not for respiratory diseases. The risk of ambulance dispatches increased by 6% (95% CI: 4%, 7%), 7% (95% CI: 5%, 9%), and 18% (95% CI: 12%, 23%) under low-intensity, severe, and extreme heatwaves, respectively. Heat exposure is associated with an increased risk of ambulance dispatches, and there is a dose-response relationship between heatwave intensity and the risk of ambulance dispatches. |
| 38 | Zhang Y, et al., 2022 [38] | Australia | Embase, Medline, PsycINFO, Scopus, Web of Science (5) | The general population | Bushfire | Mental health | The overall prevalence of long-term psychological problems in firefighters at 2–7 years ranged from 28% to 47.6%. The prevalence of some psychological issues decreased with time and was directly proportional to the level of bushfire impact. |

**Table S3.** Summary of Quality Assessment for 38 included studies

**Table S4.** Strength of evidence for climate change related risks and health outcomes

|  | | **Health outcomes** | | | | | | | | |  |  |
| --- | --- | --- | --- | --- | --- | --- | --- | --- | --- | --- | --- | --- |
|  |  | All-cause mortality/morbidity | Heat-related illnesses | Vector-borne diseases | Food-borne diseases | Water-borne diseases | Mental health disorders | Cardiovascular diseases | Respiratory diseases | Renal diseases | Injuries | Adverse birth outcomes |
| **Climate change related risks** | Heat | Sufficient | Sufficient | Sufficient | Limited | Limited | Sufficient | Sufficient | Sufficient | Sufficient | Sufficient | Limited |
|  | Bushfires (smoke) | Sufficient | - | - | - | - | Sufficient | Sufficient | Sufficient | - | Limited | Limited |
|  | Floods | Sufficient | - | Limited | Limited | Limited | Sufficient | Limited | Limited | Limited | Sufficient | Limited |
|  | Droughts | - | - | - | - | - | Sufficient | - | - | - | - | - |
|  | Cyclones | - | - | - | - | - | Lack | - | - | - | - | - |
|  | Rising sea levels | - | - | - | - | - | Lack | - | - | - | - | - |

**References**

[1] Adnan MSG, Dewan A, Botje D, Shahid S, Hassan QK. Vulnerability of Australia to heatwaves: A systematic review on influencing factors, impacts, and mitigation options. Environ Res. 2022;213:113703.

[2] Alderman K, Turner LR, Tong S. Floods and human health: a systematic review. Environ Int. 2012;47:37-47.

[3] Barros B, Oliveira M, Morais S. Continent-based systematic review of the short-term health impacts of wildfire emissions. J Toxicol Environ Health B Crit Rev. 2023:1-29.

[4] Batterham PJ, Brown K, Trias A, Poyser C, Kazan D, Calear AL. Systematic review of quantitative studies assessing the relationship between environment and mental health in rural areas. Aust J Rural Health. 2022;30:306-20.

[5] Borchers Arriagada N, Horsley JA, Palmer AJ, Morgan GG, Tham R, Johnston FH. Association between fire smoke fine particulate matter and asthma-related outcomes: Systematic review and meta-analysis. Environ Res. 2019;179:108777.

[6] Campbell S, Remenyi TA, White CJ, Johnston FH. Heatwave and health impact research: A global review. Health Place. 2018;53:210-8.

[7] Cheng J, Xu Z, Bambrick H, Prescott V, Wang N, Zhang Y, et al. Cardiorespiratory effects of heatwaves: A systematic review and meta-analysis of global epidemiological evidence. Environ Res. 2019;177:108610.

[8] Cheng J, Xu Z, Bambrick H, Su H, Tong S, Hu W. Impacts of exposure to ambient temperature on burden of disease: a systematic review of epidemiological evidence. Int J Biometeorol. 2019;63:1099-115.

[9] Chersich MF, Pham MD, Areal A, Haghighi MM, Manyuchi A, Swift CP, et al. Associations between high temperatures in pregnancy and risk of preterm birth, low birth weight, and stillbirths: systematic review and meta-analysis. BMJ. 2020;371:m3811.

[10] Daghagh Yazd S, Wheeler SA, Zuo A. Key Risk Factors Affecting Farmers' Mental Health: A Systematic Review. Int J Environ Res Public Health. 2019;16.

[11] Damtew YT, Tong M, Varghese BM, Anikeeva O, Hansen A, Dear K, et al. Effects of high temperatures and heatwaves on dengue fever: a systematic review and meta-analysis. eBioMedicine. 2023;91:104582.

[12] Damtew YT, Tong M, Varghese BM, Hansen A, Liu J, Dear K, et al. Associations between temperature and Ross River virus infection: A systematic review and meta-analysis of epidemiological evidence. Acta Trop. 2022;231:106454.

[13] Dorji T, Morrison-Saunders A, Blake D. Understanding How Community Wellbeing is Affected by Climate Change: Evidence From a Systematic Literature Review. Environ Manage. 2023;72:568-86.

[14] Evans J, Bansal A, Schoenaker D, Cherbuin N, Peek MJ, Davis DL. Birth Outcomes, Health, and Health Care Needs of Childbearing Women following Wildfire Disasters: An Integrative, State-of-the-Science Review. Environ Health Perspect. 2022;130:86001.

[15] Fatima SH, Rothmore P, Giles LC, Varghese BM, Bi P. Extreme heat and occupational injuries in different climate zones: A systematic review and meta-analysis of epidemiological evidence. Environ Int. 2021;148:106384.

[16] Faurie C, Varghese BM, Liu J, Bi P. Association between high temperature and heatwaves with heat-related illnesses: A systematic review and meta-analysis. Sci Total Environ. 2022;852:158332.

[17] Fernandez A, Black J, Jones M, Wilson L, Salvador-Carulla L, Astell-Burt T, et al. Flooding and mental health: a systematic mapping review. PLoS One. 2015;10:e0119929.

[18] Gao Y, Huang W, Yu P, Xu R, Yang Z, Gasevic D, et al. Long-term impacts of non-occupational wildfire exposure on human health: A systematic review. Environ Pollut. 2023;320:121041.

[19] Henry S, Ospina MB, Dennett L, Hicks A. Assessing the Risk of Respiratory-Related Healthcare Visits Associated with Wildfire Smoke Exposure in Children 0-18 Years Old: A Systematic Review. Int J Environ Res Public Health. 2021;18.

[20] Huang C, Barnett AG, Wang X, Vaneckova P, FitzGerald G, Tong S. Projecting future heat-related mortality under climate change scenarios: a systematic review. Environ Health Perspect. 2011;119:1681-90.

[21] Kõlves K, Kõlves KE, De Leo D. Natural disasters and suicidal behaviours: a systematic literature review. J Affect Disord. 2013;146:1-14.

[22] Liu JC, Pereira G, Uhl SA, Bravo MA, Bell ML. A systematic review of the physical health impacts from non-occupational exposure to wildfire smoke. Environ Res. 2015;136:120-32.

[23] Liu J, Varghese BM, Hansen A, Borg MA, Zhang Y, Driscoll T, et al. Hot weather as a risk factor for kidney disease outcomes: A systematic review and meta-analysis of epidemiological evidence. Sci Total Environ. 2021;801:149806.

[24] Liu J, Varghese BM, Hansen A, Xiang J, Zhang Y, Dear K, et al. Is there an association between hot weather and poor mental health outcomes? A systematic review and meta-analysis. Environ Int. 2021;153:106533.

[25] Liu J, Varghese BM, Hansen A, Zhang Y, Driscoll T, Morgan G, et al. Heat exposure and cardiovascular health outcomes: a systematic review and meta-analysis. Lancet Planet Health. 2022;6:e484-e95.

[26] Mason H, King JC, Peden AE, Franklin RC. Systematic review of the impact of heatwaves on health service demand in Australia. BMC Health Serv Res. 2022;22:960.

[27] Naish S, Dale P, Mackenzie JS, McBride J, Mengersen K, Tong S. Climate change and dengue: a critical and systematic review of quantitative modelling approaches. BMC Infect Dis. 2014;14:167.

[28] Phung D, Thai PK, Guo Y, Morawska L, Rutherford S, Chu C. Ambient temperature and risk of cardiovascular hospitalization: An updated systematic review and meta-analysis. Sci Total Environ. 2016;550:1084-102.

[29] Skinner R, Luther M, Hertelendy AJ, Khorram-Manesh A, Sørensen J, Goniewicz K, et al. A Literature Review on the Impact of Wildfires on Emergency Departments: Enhancing Disaster Preparedness. Prehosp Disaster Med. 2022;37:657-64.

[30] Tall JA, Gatton ML, Tong S. Ross River Virus Disease Activity Associated With Naturally Occurring Nontidal Flood Events in Australia: A Systematic Review. J Med Entomol. 2014;51:1097-108.

[31] Thompson R, Lawrance EL, Roberts LF, Grailey K, Ashrafian H, Maheswaran H, et al. Ambient temperature and mental health: a systematic review and meta-analysis. Lancet Planet Health. 2023;7:e580-e9.

[32] Wu Q, Yang M, Wu K, Su H, Huang C, Xu Z, et al. Abnormal ambient temperature change increases the risk of out-of-hospital cardiac arrest: A systematic review and meta-analysis of exposure types, risk, and vulnerable populations. Sci Total Environ. 2023;861:160554.

[33] Xu Z, Bambrick H, Frentiu FD, Devine G, Yakob L, Williams G, et al. Projecting the future of dengue under climate change scenarios: Progress, uncertainties and research needs. PLoS Negl Trop Dis. 2020;14:e0008118.

[34] Xu Z, Crooks JL, Davies JM, Khan AF, Hu W, Tong S. The association between ambient temperature and childhood asthma: a systematic review. Int J Biometeorol. 2018;62:471-81.

[35] Xu Z, Etzel RA, Su H, Huang C, Guo Y, Tong S. Impact of ambient temperature on children's health: a systematic review. Environ Res. 2012;117:120-31.

[36] Xu Z, Sheffield PE, Su H, Wang X, Bi Y, Tong S. The impact of heat waves on children's health: a systematic review. Int J Biometeorol. 2014;58:239-47.

[37] Xu Z, Watzek JT, Phung D, Oberai M, Rutherford S, Bach AJE. Heat, heatwaves, and ambulance service use: a systematic review and meta-analysis of epidemiological evidence. Int J Biometeorol. 2023.

[38] Zhang Y, Workman A, Russell MA, Williamson M, Pan H, Reifels L. The long-term impact of bushfires on the mental health of Australians: a systematic review and meta-analysis. Eur J Psychotraumatol. 2022;13:2087980.
